# Comparative study of wave-front aberration and corneal Asphericity after SMILE and LASEK for myopia: a short and long term study

**DOI:** 10.1186/s12886-019-1084-3

**Published:** 2019-03-20

**Authors:** Manrong Yu, Minjie Chen, Wangyuan Liu, Jinhui Dai

**Affiliations:** 1 Department of Ophthalmology, Eye and ENT Hospital, Fudan University, No. 83 Fenyang Road, Shanghai, 200031 China; 2 NHC Key Laboratory of Myopia (Fudan University) and Laboratory of Myopia, Chinese Academy of Medical Sciences, Shanghai, China

**Keywords:** Small incision Lenticule extraction, Laser-assisted subepithelial Keratomileusis, Corneal refractive power, Corneal asphericity, Wave-front aberration

## Abstract

**Background:**

The study compares the wave-front aberration and corneal asphericity from multiple perspectives after Small Incision Lenticule Extraction and Laser-assisted Subepithelial Keratomileusis for mild to moderate myopia in a short and long time period.

**Methods:**

This prospective and comparative study included 32 eyes in the SMILE group, with a mean spherical equivalent (SE) of − 4.1 ± 0.9D and 32 eyes in the LASEK group, with a mean SE of − 3.7 ± 1.0D. Visual acuity, refractive error, wave-front aberration, corneal Q value and corneal refractive power were analyzed pre-, 3 months and 3 years post-operatively.

**Results:**

There was no significant difference in refractive error, wave-front aberration, corneal Q value and corneal refractive power before treatment. Three months postoperative, Q value within 6 mm (SMILE: 0.46 ± 0.27, LASEK: 0.63 ± 0.28, *p* = 0.02), the relative peripheral corneal power (5-8 mm: *p* < 0.05), change of higher order aberration (SMILE: 0.10 ± 0.16, LASEK: 0.24 ± 0.20, *p* = 0.004) and spherical aberration (SA, SMILE: -0.07 ± 0.30, LASEK: -0.41 ± 0.40, *p* < 0.001) were significantly lower in the SMILE than in LASEK group. The visual acuity, refractive error, coma, peripheral Q value, central corneal power had no significant difference between the two groups. Three years post-operation, the corneal power distribution results and SA were similar to that of 3-month, while the Q value had no significant difference between the two groups.

**Conclusion:**

In the early stage after SMILE, the HOAs was lower, the corneal refractive power from central to periphery was more uniform than after LASEK; and in the long-term run, SMILE still preceded LASEK in the corneal asphericity and aberration.

**Electronic supplementary material:**

The online version of this article (10.1186/s12886-019-1084-3) contains supplementary material, which is available to authorized users.

## Background

Small Incision Lenticule Extraction (SMILE) was first introduced in 2011 for the correction of myopia, and the safety and efficacy of SMILE has been approved by previous studies [1–3]. It has been demonstrated to produce less coma and spherical aberration than femtosecond laser in situ keratomileusis (FS-LASIK) [4–6]. It may be dependent on the precision of the lenticule creation of the femtosecond laser [7]. Furthermore, SMILE does not create a flap and preserves almost original anterior corneal structure, which may contribute to overall better visual quality. Another kind of nearly flapless refractive surgery, laser-assisted subepithelial keratomileusis (LASEK), creates no stromal-flap-related higher order aberration (HOA) either. It was reported that patients had better quality of vision after LASEK than LASIK [8]. With the same excimer laser, non-flap mode can account for the majority difference.

Unlike the femtosecond laser, the energy efficiency of excimer laser is not uniform from central to peripheral and thereby the ablation rate decrease towards the periphery, which may induce the HOA, resulting from the peripheral ablation depth not matching the design [9]. Without the influence of the corneal flap, the comparison between the SMILE and LASEK is more like the comparison of femtosecond laser and excimer laser, and the different corneal-tissue-removing pattern, and which treatment inducing less asphericity change and better visual quality remained unclear. The corneal ashphericity could be represented by the corneal Q value, and could also be reflected by the change of corneal power from center to periphery [10, 11].

To our knowledge, there has been no study comparing the change of corneal asphericity and its relationship with visual quality after SMILE and LASEK. In this study, we compared and analyzed the corneal asphericity change following SMILE and LASEK with the Pentacam, to interpret the difference of visual quality after the two procedures.

## Methods

### Participants

This prospective, comparative study enrolled 64 consecutive patients (64 eyes) of whom 32 patients (17 men and 15 women) underwent SMILE and 32 patients (15 men and 17 women) underwent LASEK. Patient inclusion criteria include an age of 18 to 40 years old with a stable refractive error for at least 2 years prior (<− 0.25 D change per year), spherical equivalent from − 1.25D to − 6.00D, and a minimum estimated residual stromal bed thickness of 280 μm. Patients with a corrected distance visual acuity (CDVA) less than 20/25 or those with systemic or localized ocular disease were excluded. All surgeries were conducted in the Eye and ENT Hospital of Fudan University, Shanghai, P.R. China between January 2014 and May 2014, and patients chose the type of surgery voluntarily without the controls of doctors after understanding the cons and pros of each kind of surgery.

The institutional review board of The Ethics Committee of the Eye and ENT Hospital affiliated to Fudan University approved this study prospectively. Written informed consent was obtained from all patients before surgery. All subjects were treated in accordance with the tenets of the Declaration of Helsinki.

### Preoperative examination

All patients received the routine preoperative ophthalmologic examination, including uncorrected distance visual acuity (UDVA), corrected distance visual acuity (CDVA), intraocular pressure (non-contact tonometer), refraction (manifest and cycloplegic) and corneal tomography. A detailed anterior and posterior segment examination was performed via a slit lamp.

Corneal tomography was performed using Rotating Scheimpflug imaging technology (Pentacam; Oculus, Wetzlar, Germany). Participants were instructed to keep both eyes open widely and look directly at the fixation target when measuring corneal tomography, and the data could be accepted when the image quality was OK. The wave-front aberration for a 6 mm pupil diameter was measured by the WASCA (WASCA wavefront analyzer; Carl Zeiss Meditec AG, Jena, Germany).

### Surgical techniques

All surgical procedures were performed after careful cleaning of the conjunctival sac and application of three drops of topical anesthesia (0.4% oxybuprocaine hydrochloride Santen Pharmaceutical Co., Ltd., Osaka, Japan).

The SMILE procedure was performed with VisuMax femtosecond laser system (Carl Zeiss Meditec, Jena, Germany), and the repetition rate and pulse energy was 500 kHz and 130 nJ, respectively. The cap thickness and diameter was set to 120 μm and 7.5 mm, and the lenticule diameter was 6.5 mm (no astigmatism) or 6.6 mm (with astigmatism). The refractive lenticule of the stromal corneal tissue was extracted after created through a 2 mm-side cut located at the 12’o-clock position.

In terms of LASEK, corneal stromal tissue ablation was performed using the Mel 80 excimer laser (Carl Zeiss Meditec) with standard ablation protocol over an optical diameter ranging from 6.25 mm to 6.75 mm, surrounded by a 1.5 mm transition zone, after a corneal epithelial flap was created with 20% ethanol-aqueous solution and was peeled back with a crescent blade (Model 52424A; 66vision Tech Co., Ltd., Suzhou, China). No patient was treated with mitomycin C as the refractive error was under − 6.00D. The epithelial flap was then repositioned and a bandage contact lens (ACUVE OASYS, Johnson & Johnson, Inc) was inserted for 7 days. The detailed surgical procedure can be found in a previous publication [12].

A routine postoperative medication consisted of topical fluorometholone 0.1%, used initially six times a day and tapered for a period of 30 days after SMILE and 60 days after LASEK, topical levofloxacin 0.5%, used four times a day for a week, and artificial tears, used throughout the 90-day period postoperatively.

### Postoperative ophthalmologic examinations

Follow-up examinations, including UCVA, refraction, slit-lamp examination, wave-front aberrations and corneal tomography, were scheduled at 3 months and 3 years postoperatively. The postoperative examinations were done by two independent technicians, who were blind to the surgical procedures patients received.

#### Wave-front aberrations

The corneal wavefront aberrations were analyzed for a standardized pupil diameter of 6 mm. The Zernike coefficients of vertical trefoil (${Z}_3^{-3}$), horizontal trefoil (${Z}_3^3$), vertical coma (${Z}_3^{-1}$), horizontal coma (${Z}_3^1$), and spherical aberration (SA) (Z^0^_4_) were analyzed [6]. In addition, the root mean square (RMS), expressed as coma ($\sqrt{{\left({Z}_3^1\right)}^2+{\left({Z}_3^{-1}\right)}^2}$), trefoil ($\sqrt{{\left({Z}_3^3\right)}^2+{\left({Z}_3^{-3}\right)}^2}$) and HOAs were calculated because they are clinically significant in determining visual quality.

#### Corneal Asphericity

The corneal asphericity can be reflected by the corneal Q value, distribution of sagittal front corneal refractive power (SAG) and total corneal refractive power (TCRP), which can be provided by the Pentacam corneal tomography. The Pentacam reported Q values, centered on the corneal apex with increasing diameters at 1.0 mm steps from 6 mm to 10 mm. The SAG and TCRP were presented in the power distribution display in Pentacam, presented in diameter from 1 mm to 8 mm, centered on corneal apex at 1 mm steps [13]. The best-fit sphere diameter was set to 8 mm in all cases. The SAG and TCRP on pupil center were also recorded. The surgically induced change in corneal refractive power, SAG or TCRP, was calculated as ∆SAG = Km (SAG)_pre_ − Km (SAG)_post_, ∆TCRP=Km (TCRP)_pre_ − Km (TCRP)_post_ (Eq. 1) where Km was the mean corneal power at the concentric of certain distance from the center, m ranged from 1 to 8 mm at 1 mm steps. The relative peripheral corneal power was calculated as periSAG = Km (SAG) − K0(SAG), periTCRP = Km (TCRP) –K0 (TCRP) (Eq. 2) where Km represented the same value as in Eq. 1, and K0 was the corneal power at the corneal apex.

### Statistical analysis

To have an 80% chance of detecting a 0.2 μm difference between SMILE and LASEK with 0.25 μm standard deviation (SD) in spherical aberration at the *p* = 0.05 level (two- sided), 24 cases were required. Considering the loss of following-up, we recruited more than 29 patients in each group for the study. Data from the right eye of each patient was enrolled for statistical analysis. All statistical analyses were performed using SPSS 20.0 software (SPSS, Inc., Armonk, NY) and reported as mean ± standard deviation (SD). Student’s t-test was used to compare normally distributed data between the two groups. The Mann-Whitney rank-sum test was used to analyze non-normally distributed data. Pearson’s linear correlation was used to investigate the linear relationships between the change of SA and corneal refractive power or corneal asphericity. In aberration analysis, *p* value less than 0.05 was considered statistically significant, while in corneal refractive power analysis, *p* < 0.006 was considered statistically significant (corrected for multiple comparisons (Bonferroni–Holm)).

## Results

All surgeries were uneventful and there was no specific intraoperative or post-operative complication during the study. All patients in both groups completed the pre- and post-operative examinations. Pre-operative data were comparable between the two groups, which were summarized and presented as mean standard deviation in Table 1 in detail. The lenticule diameter in SMILE was 6.6 mm in 29 eyes and 6.5 mm in 3 eyes, averaging (6.59 ± 0.03) mm; the optical diameter in LASEK was 6.75 mm in 3 eyes, 6.5 mm in 28 eyes and 6.25 mm in one eye, averaging (6.52 ± 0.09) mm. The post-3-year refractive error (SMILE: -0.30 ± 0.42D, LASEK: -0.19 ± 0.41D) and uncorrected visual acuity (SMILE: -0.10 ± 0.05logMAR, LASEK: -0.11 ± 0.09 logMAR) were comparative between the two groups (*p* > 0.05).

**Table 1:** Preoperative Demographic Data^a^

	SMILE (*N* = 32)	LASEK (N = 32)	t/Z^#^	*P* value
Age (y)	24.2 ± 4.5	24.9 ± 5.3	− 0.57	0.57
Male: female	17:15	15:17	−0.5	0.62
Spherical Equivalent (D)	−4.1 ± 0.9	−3.7 ± 1.0	−1.58	0.12
Scotopic Pupil Diameter (mm)	7.2 ± 0.5	7.4 ± 0.5	−1.6	0.11
Central Corneal Thickness (μm)	553.0 ± 27.2	544.0 ± 35.0	1.15	0.25
Ablation depth (μm)	92.7 ± 11.7	76.8 ± 16.8	4.43	< 0.001*

a Data are given as mean ± standard deviation

*p < 0.05

# t: Student’s t test; Z: Mann-Whitney Test

### Corneal asphericity

#### Corneal Q value

**Fig. 1:**
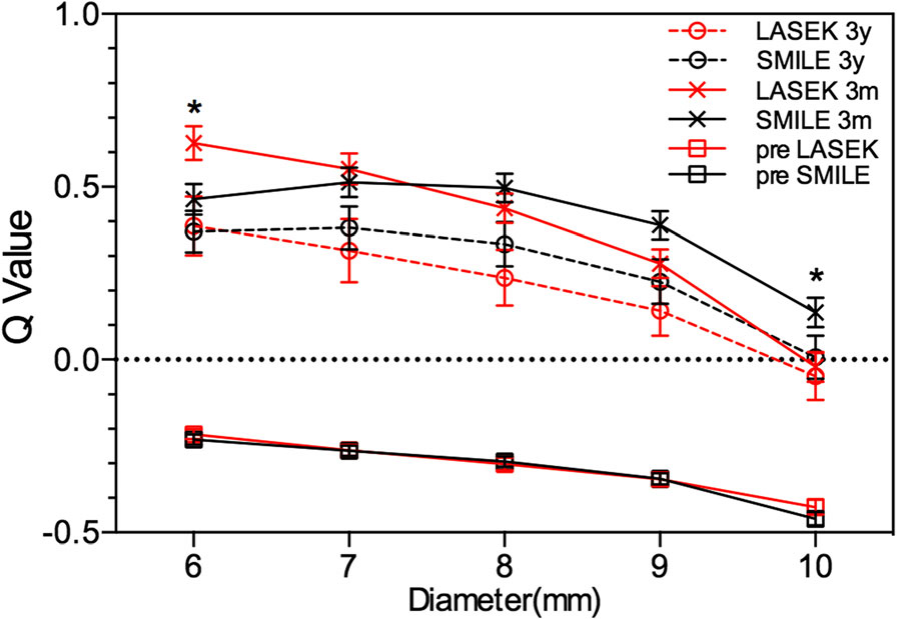
The comparison of Q value (corneal asphericity) between SMILE and LASEK from 6-to 10-mm pre-, three months (**a**) and three years (**b**) post-operatively. The horizontal axis corresponds to the diameter from 6.0 to 10.0 mm in 1.0-mm increments, centered on the corneal apex; the vertical axis corresponds to Q value. * *p* < 0.05 = statistically significant

**Fig. 2:**
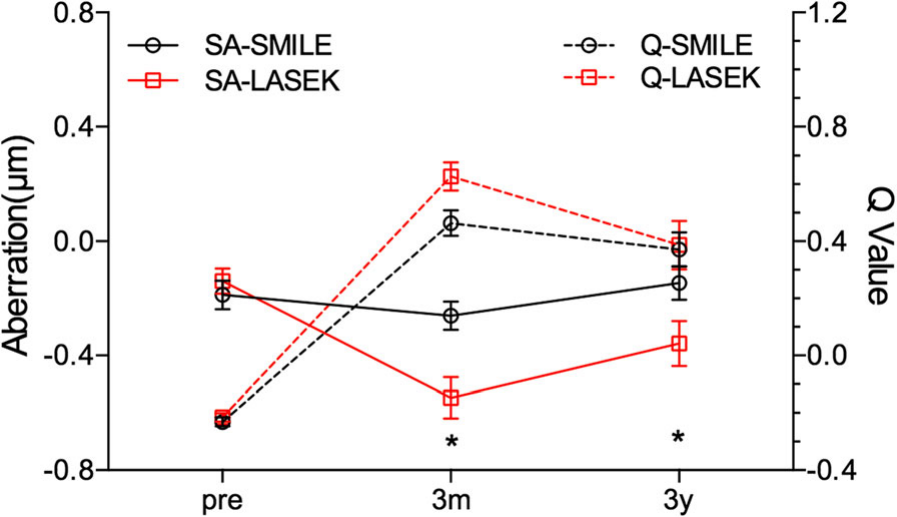
The comparison of spherical aberration (SA, left y-axis) and corneal 6 mm-diameter-Q value (right y-axis) between SMILE and LASEK at pre-, three months and three years post-operatively. The horizontal axis corresponds to the time point of follow-up; the left vertical axis corresponds to spherical aberration (SA), the right vertical axis represents the Q value. * *p* < 0.05 = statistically significant

The Q value was comparable between the two groups before treatment, and the larger the calculation diameter, the smaller the Q value, as shown in Fig. 1. The anterior corneal Q value in both groups changed from negative (corresponding to a prolate corneal shape) to positive (corresponding to an oblate corneal shape) after surgery. The Q value within 6 mm in SMILE was lower than in LASEK (SMILE: 0.46 ± 0.27, LASEK: 0.63 ± 0.28, t = − 2.47, *p* = 0.02) 3 months post operation (Fig. 1), while it had no significant difference 3 years later. The 6-mm-Q value decreased significantly from 3 months to 3 years after LASEK (Fig. 2), while it didn’t change remarkably during that time after SMILE.

#### Corneal refractive power

The preoperative SAG and TCRP were comparable between the two groups, as shown in Table 2. An expected finding is that the larger the observed zone diameter, the higher the total corneal refractive power, and the smaller the sagittal front corneal power.

**Table 2:** The corneal refractive power distribution

	pre SMILE	pre LASEK	SMILE 3 m	LASEK 3 m	*p* value	SMILE 3y	LASEK 3y	*p* value
Sagittal corneal refractive power								
k1	42.89 ± 1.21	42.85 ± 1.38	39.07 ± 1.25	39.52 ± 1.91	0.26	39.23 ± 1.08	40.09 ± 1.65	0.02
k2	42.85 ± 1.19	42.84 ± 1.38	39.01 ± 1.24	39.46 ± 1.87	0.26	39.12 ± 1.10	39.97 ± 1.61	0.02
k3	42.80 ± 1.17	42.81 ± 1.38	38.93 ± 1.27	39.38 ± 1.81	0.25	39.01 ± 1.17	39.90 ± 1.58	0.01
k4	42.75 ± 1.16	42.78 ± 1.37	38.87 ± 1.30	39.41 ± 1.76	0.17	38.96 ± 1.17	40.03 ± 1.57	0.003*
k5	42.69 ± 1.15	42.74 ± 1.38	38.86 ± 1.30	39.52 ± 1.71	0.08	39.09 ± 1.10	40.33 ± 1.54	< 0.001*
k6	42.61 ± 1.14	42.68 ± 1.37	38.94 ± 1.28	39.75 ± 1.66	0.03	39.46 ± 1.01	40.81 ± 1.53	< 0.001*
k7	42.50 ± 1.14	42.57 ± 1.37	39.11 ± 1.23	40.06 ± 1.61	0.01	40.00 ± 0.98	41.26 ± 1.53	< 0.001*
k8	42.32 ± 1.13	42.42 ± 1.37	39.39 ± 1.17	40.33 ± 1.57	0.008	40.64 ± 1.00	41.49 ± 1.52	0.01
Total corneal refractive power								
k1	42.02 ± 1.20	41.94 ± 1.36	37.45 ± 1.29	38.03 ± 1.95	0.16	37.78 ± 1.14	38.72 ± 1.68	0.01
k2	42.03 ± 1.19	41.96 ± 1.37	37.41 ± 1.29	37.97 ± 1.92	0.17	37.71 ± 1.16	38.64 ± 1.65	0.01
k3	42.06 ± 1.18	42.01 ± 1.37	37.38 ± 1.31	37.95 ± 1.88	0.16	37.65 ± 1.23	38.67 ± 1.59	0.005*
k4	42.14 ± 1.15	42.12 ± 1.37	37.40 ± 1.36	38.05 ± 1.82	0.11	37.82 ± 1.25	39.01 ± 1.59	0.001*
k5	42.26 ± 1.15	42.27 ± 1.41	37.52 ± 1.37	38.32 ± 1.77	0.05	38.24 ± 1.20	39.71 ± 1.59	< 0.001*
k6	42.44 ± 1.16	42.46 ± 1.42	37.79 ± 1.34	38.80 ± 1.73	0.01	39.14 ± 1.09	40.77 ± 1.67	< 0.001*
k7	42.62 ± 1.18	42.67 ± 1.44	38.26 ± 1.29	39.43 ± 1.71	0.003*	40.44 ± 1.10	41.99 ± 1.74	< 0.001*
k8	42.82 ± 1.21	42.74 ± 1.42	38.94 ± 1.24	40.15 ± 1.69	0.002*	42.12 ± 1.19	43.23 ± 1.82	0.005*

*p < 0.006 = significantly different, corrected for multiple comparisons (Bonferroni–Holm)

Three months postoperatively, the SAG had no significant difference between SMILE and LASEK group (see Table 2); and ∆SAG was greater at 5- to 8-mm in SMILE than in LASEK group (see Additional file 1, Fig. 3A), correspondingly. The 6-8 mm periSAG was significantly lower in SMILE than in LASEK, the difference was about 0.5D (see Additional file 2, Fig. 3C).

**Fig. 3:**
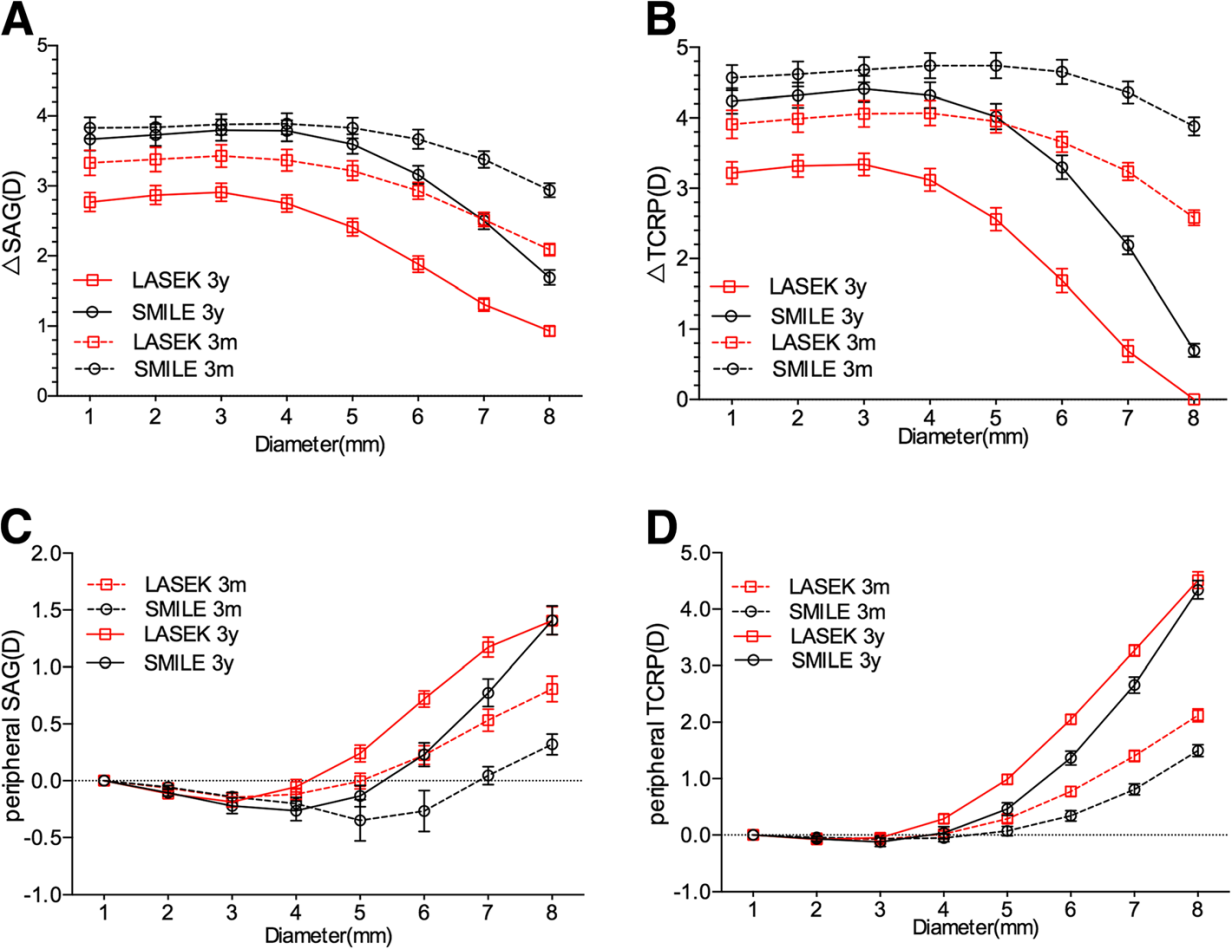
(**a**) Comparison of surgically induced change in SAG (∆SAG) between SMILE and LASEK at 3-month and 3-year post operation. The horizontal axis corresponds to the diameter from 1.0 to 8.0 mm in 1.0-mm increments, centered on the corneal apex; the vertical axis stands for surgically induced change of SAG. (**b**) Comparison of surgically induced change in TCRP (∆TCRP) between SMILE and LASEK at 3-month and 3-year post operation. The horizontal axis is the same value in (**a**); the vertical axis stands for surgically induced change of TCRP. (**c**) Comparison of relative peripheral SAG between SMILE and LASEK at 3-month and 3-year post operation. The horizontal axis is the same parameter in (**a**); the vertical axis stands for the relative peripheral SAG. (**d**) Comparison of relative peripheral TCRP between SMILE and LASEK at 3-month and 3-year post operation. The horizontal axis is the same parameter in (**a**); the vertical axis stands for the relative peripheral TCRP

The results of TCRP distribution were similar to the SAG results, while TCRP at 7- and 8-mm were lower in SMILE than in LASEK (see Table 2); and ∆TCRP from 5- to 8-mm were larger in SMILE than in LASEK group (see Additional file 1, Fig. 3B). Similar to SAG, the 6-8 mm periTCRP was significantly lower in SMILE than in LASEK, and the more peripheral, the larger the difference (see Additional file 2, Fig. 3D).

Three years post-operation, the SAG at 4-7 mm in SMILE were lower than that in LASEK group (see Table 2); and ∆SAG from center to periphery in SMILE were greater than LASEK (see Additional file 1, Fig. 3A), correspondingly. The significant difference of periSAG between SMILE and LASEK only appeared at 5-and 6-mm circle (see Additional file 2, Fig. 3C).

The difference of the TCRP between LASEK and SMILE were greater at 3 years than 3 months post-operation. The TCRP at 3-8 mm in SMILE were lower than that in LASEK (Table 2), while ∆TCRP at all area had significant difference between SMILE and LASEK (Additional file 1, Fig. 3B). Similar to SAG, periTCRP at 5-7 mm were lower in SMILE than in LASEK (Additional file 2, Fig. 3D).

### Wave-front aberrations

The RMS of aberrations pre-operatively, including total aberrations, HOA, SA, trefoil and coma, was not significantly different between the SMILE and LASEK groups (Table 3.). Three months post-operation, HOA and SA in SMILE were lower than in LASEK (HOA: *p* < 0.001; SA: *p* = 0.002). Three years after the procedure, HOA and SA remained lower in SMILE than in LASEK (HOA: *p* = 0.03, SA: *p* = 0.04), moreover, vertical coma was significantly lower in SMILE than in LASEK (*p* = 0.003).

**Table 3:** Comparison of wave-front aberration in the two groups pre and post surgery

	pre SMILE	pre LASEK	SMILE 3 m	LASEK 3 m	*p* value	SMILE 3y	LASEK 3y	*p* value
Vertical coma	0.07 ± 0.26	0.01 ± 0.26	0.34 ± 0.45	0.61 ± 0.67	0.06	0.13 ± 0.55	0.58 ± 0.62	0.003*
Horizontal coma	−0.12 ± 0.49	− 0.16 ± 0.49	0.34 ± 0.59	0.20 ± 0.59	0.34	0.10 ± 0.55	0.15 ± 0.47	0.68
Vertical trefoil	−0.02 ± 0.26	0.01 ± 0.26	−0.03 ± 0.25	0.11 ± 0.28	0.04*	− 0.04 ± 0.29	0.02 ± 0.36	0.45
Horizontal trefoil	−0.14 ± 0.31	− 0.09 ± 0.43	0.00 ± 0.30	− 0.03 ± 0.46	0.83	− 0.18 ± 0.29	−0.08 ± 0.37	0.26
Spherical aberration	−0.19 ± 0.28	−0.14 ± 0.25	− 0.26 ± 0.28	−0.55 ± 0.41	0.002*	− 0.15 ± 0.33	−0.36 ± 0.45	0.04*
Coma	0.48 ± 0.30	0.50 ± 0.28	0.73 ± 0.48	0.88 ± 0.64	0.29	0.70 ± 0.57	0.88 ± 0.57	0.21
Trefoil	0.36 ± 0.24	0.45 ± 0.24	0.33 ± 0.20	0.46 ± 0.29	0.04*	0.36 ± 0.25	0.43 ± 0.28	0.30
HOA	0.29 ± 0.11	0.29 ± 0.11	0.39 ± 0.13	0.53 ± 0.17	< 0.001*	0.47 ± 0.15	0.55 ± 0.11	0.03*

HOA: Higher order aberration

3 m: three months post operation

3y: three years post operation

*p < 0.05 significantly different

In terms of the change of aberration, 3 months post-operation, the change of vertical coma, SA and HOA was significantly lower in SMILE than in LASEK (vertical coma: *p* = 0.01; SA: p < 0.001; HOA: *p* = 0.004), while only change of vertical coma and SA remained smaller in SMILE 3 years postoperative (*p* = 0.001; *p* = 0.009). In addition, the change from 3 months to 3 years had no significant difference between the two groups (see Additional file 3).

### Correlation between corneal asphericity and spherical aberration

**Fig. 4:**
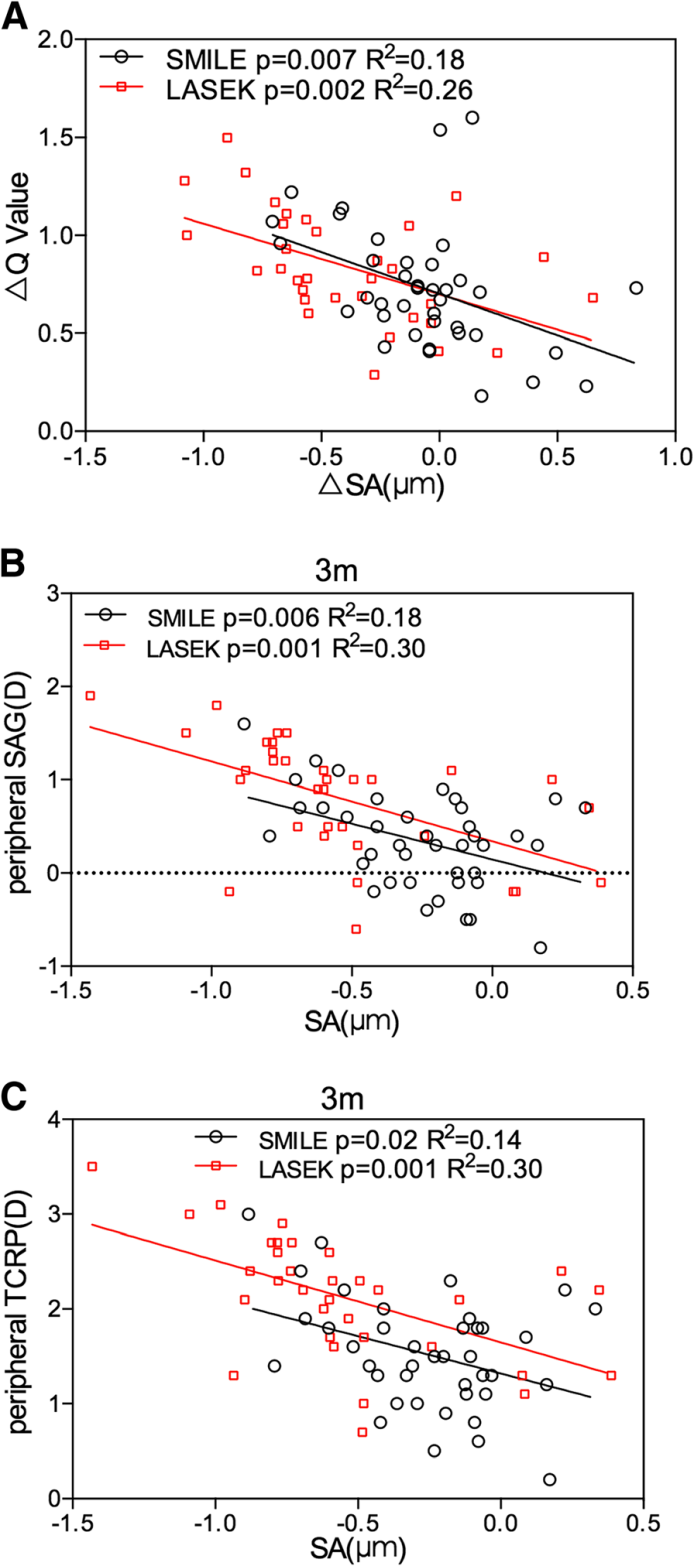
(**a**) Correlation of surgically induced spherical aberration (∆SA, x-axis) and change of corneal asphericity (∆Q value, y-axis) at 3-month post-operation in SMILE and LASEK. (**b**) Correlation of 3-month postoperative spherical aberration (SA, x-axis) and relative peripheral sagittal corneal refractive power (peripheral SAG, y-axis) in SMILE and LASEK. (**c**) Correlation of 3-month postoperative spherical aberration (SA, x-axis) and relative peripheral total corneal refractive power (peripheral TCRP, y-axis) in SMILE and LASEK

SA and Q value changed oppositely after treatment (Fig. 2), suggesting that there was negative correlation between them. As in Fig. 4A, the change of SA negatively correlated with the change of Q value at 3 months post operation in both groups (*p* < 0.05). The preoperative Q value negatively correlated with SA (SMILE: p = 0.003; LASEK: p = 0.002), and the preoperative and 3-month-postoperative peripheral corneal power negatively correlated with SA (Fig. 4B&C), however, the 3-year peripheral corneal power didn’t follow this rule (TCRP& SA: SMILE: *p* = 0.26; LASEK: *p* = 0.08).

## Discussion

SMILE and LASEK are now commonly used for correcting myopia and astigmatism, and the two flapless procedures were proven to be safe and effective by previous studies [2, 14, 15]. Though SMILE and LASEK demonstrate priority to the flap procedure in visual quality, no study concerns the disparity between them. We found in the study that the HOAs was lower, the corneal refractive power from central to periphery was more uniform than after LASEK at 3 months postoperatively. It was consistent with our previous observation showed that the HOAs, especially spherical aberration (SA), was lower 3 months following SMILE than LASEK [12]. While in the long-term run, the corneal asphericity and aberration was similar after the two treatments in current study.

The change of corneal asphericity is in relationship with visual quality after refractive surgery. With the measurement diameter of 9 mm and the analysis diameter of 8 mm, Pentacam can provide us a comprehensive data to analyze the corneal asphericity. The current study analyzed corneal asphericity after SMILE and the Q value from central to peripheral, and observed that the Q value within 6 mm was smaller in SMILE than LASEK. As the optical zone diameter was 6.25–6.75, the Q value within 7 mm was the most important. The 3-year results demonstrated that the Q value after LASEK decreased from 3 month post-operation, and it was not significantly different from SMILE. The smaller the Q value, the more prolate the corneal shape, which agrees with the lower SA after SMILE than LASEK at an early stage after surgery. When having a long time recovery, the Q value in LASEK decreased to the level of that in SMILE, and the SA had no significant difference between the two groups, either. Kamiya et al. reported that the Q value changed from − 0.23 ± 0.09 to 0.42 ± 0.30 and from − 0.20 ± 0.08 to 0.65 ± 0.30 following FLEx and wave-front guided LASIK, respectively [16]. Moreover, the Q value was smaller after FLEx than LASIK. The results were similar with the change of Q value following SMILE and LASEK in the current study. The reason why SMILE had smaller Q value and better corneal asphericity may be that relative peripheral corneal power was different beyond 5 mm-diameter between SMILE and LASEK. The change of corneal power at peripheral was different between the two groups, and the more peripheral, the larger the difference, suggesting that the peripheral corneal tissue removed by femtosecond laser was more than excimer laser, which may result from the difference of energy efficiency in the periphery between the two instrument [17] and the transition zone design in LASEK procedure.

SAG is calculated by considering the cornea as a single refractive sphere and is free from the thickness of cornea and the depth of anterior chamber, and it’s good at describing the anterior corneal refractive power [18]. The peripheral and relative peripheral SAG was significantly lower following SMILE than LASEK in our study, meaning that the peripheral cornea was flatter in SMILE. In addition, the peripheral surgically induced corneal power change (∆SAG) was greater in SMILE than LASEK, indicating that the peripheral corneal power change was closer to the central power change. This may be due to that SMILE extracted the lenticule from the corneal stroma, which was designed uniform and easily to be extracted, leading to a more hyperopic peripheral defocus and lower Q value simultaneously. Also, the anterior corneal surface remained almost unchanged, preserving large amount of original corneal structure. However, LASEK removes the Bowman’s layer and the front corneal stroma, followed by the corneal epithelialization, which changes the anterior corneal surface and the refractive power distribution.

The calculation of TCRP is based on ray tracing, and corneal thickness and curvatures of both the anterior and posterior corneal surfaces are used in the calculations, which reflects the realistic corneal refractive power [19]. Similar to the SAG results, the peripheral and relative peripheral TCRP was lower in SMILE than LASEK, especially in the early stage. The difference of peripheral corneal power between SMILE and LASEK was significant, despite that the observed variation increased with increasing measurement diameter. While in the current study, TCRP in the periphery was lower post-SMILE than LASEK, the 5-8 mm relative peripheral TCRP was lower 3 months after SMILE correspondingly, and ∆TCRP from center to periphery was all higher in SMILE. This may have relevance to the transition zone outside the optical zone in LASEK procedure but not in SMILE. The difference of peripheral defocus may also result from the attenuation of excimer laser on peripheral corneal tissue, leading to the ablation depth not matching the design [20, 21]. At the three-year follow-up, the TCRP in the periphery was still lower in SMILE than LASEK. ∆TCRP decreased in both SMILE and LASEK, and the more peripheral, the larger the decease, indicating that the cornea regressed in a long term after both treatments, especially the peripheral cornea, returning to the original corneal power. This phenomenon suggests that the peripheral corneal has greater tendency to regress than the central corneal, especially in LASEK group. That’s why the 8-mm relative peripheral corneal power had no significant difference between the two groups at 3-year follow-up.

Also, we observed that the change of HOAs, especially spherical aberration, were lower after SMILE than LASEK at an early stage and after a long-term follow-up, which was consistent with the corneal refractive power distribution results but not matching the 3-year Q value. The averaged scotopic pupil diameter in the current study was larger than 7 mm, and the peripheral (≥7 mm) corneal power was lower after SMILE than LASEK. When correlating the aberration and the corneal shape change, we can speculate that the difference of HOAs between SMILE and LASEK was almost entirely result from the difference in corneal deformation. In addition, the corneal power distribution was more sensitive in association with the spherical aberration than the Q value. An interesting finding is that the change of vertical coma remained lower in SMILE than in LASEK from short to long-term observation, which may be due to the mild decentration during the laser ablation [22, 23]. However, in Reinstein et al. study, slightly larger offset observed in SMILE compared to LASIK [24]. So the comparison of decentration between SMILE and LASEK needs further investigation. A limitation of the study was that the postoperative subjective symptoms of the patients were not recorded during follow-up, which could be substantiated the objective readings and warrants further study.

## Conclusion

In conclusion, the wave-front aberration, especially the spherical aberration, changed less after SMILE than LASEK. In addition, the Q value was smaller and the refractive power was more uniform from central to peripheral after SMILE than LASEK, indicating that the femtosecond laser and excimer laser are different in terms of working patterns and the removal and maintenance of corneal tissue, which can result in a different corneal shape post-operatively.

## Additional files

Additional file 1: The change of corneal refractive power from pre operation. This table shows the comparison of the change of corneal refractive power (preoperative-postoperative) three months and three years postoperatively between SMILE and LASEK, including Sagittal corneal refractive power from preoperative and Total corneal refractive power from preoperative. (DOCX 20 kb)

Additional file 2: Relative peripheral corneal refractive power distribution. This table shows the comparison of the relative peripheral corneal refractive power distribution ((peripheral-center)/center) before and after surgeries between SMILE and LASEK, including Sagittal corneal refractive power from preoperative and Total corneal refractive power from preoperative. (DOCX 19 kb)

Additional file 3: Comparison of change in wave-front aberration in the two groups post surgery. This table shows the comparison of the change in wave-front aberration (postoperative-preoperative) between SMILE and LASEK post surgery. (DOCX 18 kb)

## Abbreviations

CDVA: Corrected distance visual acuity
HOA: Higher Order Aberration
LASEK: Laser-assisted Subepithelial Keratomileusis
SA: Spherical aberration
SAG: Sagittal front corneal refractive power
SMILE: Small Incision Lenticule Extraction
TCRP: Total corneal refractive power
