# Supplementary material for: Comparative study of wave-front aberration and corneal Asphericity after SMILE and LASEK for myopia: a short and long term study
Source: BMC Ophthalmol. 2019 Mar 20;19:80. doi: 10.1186/s12886-019-1084-3 (PMC6425702; doi:10.1186/s12886-019-1084-3)
Supplement: Supplementary file 1 — The change of corneal refractive power from pre operation. This table shows the comparison of the change of corneal refractive power (preoperative-postoperative) three months and three years postoperatively between SMILE and LASEK, including Sagittal corneal refractive power from preoperative and Total corneal refractive power from preoperative. (DOCX 20 kb) [file 12886_2019_1084_MOESM1_ESM.docx]

Additional file 1. The change of corneal refractive power from pre operation

|  | SMILE 3m | LASEK 3m | *p* value | SMILE 3y | LASEK 3y | *p* value |
| --- | --- | --- | --- | --- | --- | --- |
| Sagittal corneal refractive power from preoperative | | | | | | |
| k1 | 3.83±0.86 | 3.33±1.01 | 0.04 | 3.67±0.93 | 2.77±0.76 | <0.001* |
| k2 | 3.84±0.84 | 3.38±0.98 | 0.05 | 3.73±0.88 | 2.87±0.77 | <0.001* |
| k3 | 3.88±0.84 | 3.43±0.90 | 0.05 | 3.80±0.86 | 2.91±0.73 | <0.001* |
| k4 | 3.89±0.85 | 3.37±0.87 | 0.02 | 3.79±0.85 | 2.75±0.70 | <0.001* |
| k5 | 3.83±0.83 | 3.22±0.79 | 0.003* | 3.60±0.78 | 2.41±0.70 | <0.001* |
| k6 | 3.67±0.78 | 2.93±0.68 | 0.0001* | 3.16±0.73 | 1.88±0.66 | <0.001* |
| k7 | 3.38±0.68 | 2.52±0.58 | <0.001* | 2.50±0.67 | 1.31±0.53 | <0.001* |
| k8 | 2.94±0.56 | 2.09±0.50 | <0.001* | 1.69±0.60 | 0.93±0.39 | <0.001* |
| Total corneal refractive power from preoperative | | | | | | |
| k1 | 4.57±1.01 | 3.91±1.12 | 0.02 | 4.24±1.11 | 3.22±0.90 | <0.001* |
| k2 | 4.62±1.00 | 3.99±1.09 | 0.02 | 4.32±1.04 | 3.32±0.90 | <0.001* |
| k3 | 4.68±1.01 | 4.06±1.05 | 0.02 | 4.41±1.05 | 3.34±0.89 | <0.001* |
| k4 | 4.74±1.02 | 4.07±1.00 | 0.01 | 4.32±1.03 | 3.12±0.91 | <0.001* |
| k5 | 4.74±1.03 | 3.95±0.91 | 0.002* | 4.02±1.01 | 2.56±0.92 | <0.001* |
| k6 | 4.65±0.97 | 3.66±0.82 | <0.001* | 3.30±1.04 | 1.69±0.94 | <0.001* |
| k7 | 4.36±0.87 | 3.24±0.71 | <0.001* | 2.19±1.05 | 0.69±0.90 | <0.001* |
| k8 | 3.88±0.73 | 2.58±0.62 | <0.001* | 0.70±1.16 | -0.50±0.80 | <0.001* |

* p<0.006 significantly different
