# Supplementary material for: Comparative study of wave-front aberration and corneal Asphericity after SMILE and LASEK for myopia: a short and long term study
Source: BMC Ophthalmol. 2019 Mar 20;19:80. doi: 10.1186/s12886-019-1084-3 (PMC6425702; doi:10.1186/s12886-019-1084-3)
Supplement: Supplementary file 2 — Relative peripheral corneal refractive power distribution. This table shows the comparison of the relative peripheral corneal refractive power distribution ((peripheral-center)/center) before and after surgeries between SMILE and LASEK, including Sagittal corneal refractive power from preoperative and Total corneal refractive power from preoperative. (DOCX 19 kb) [file 12886_2019_1084_MOESM2_ESM.docx]

Additional file 2. Relative peripheral corneal refractive power distribution

|  | | pre SMILE | | pre LASEK | | SMILE 3m | | LASEK 3m | | *p* value | | SMILE 3y | | LASEK 3y | | *p* value |
| --- | --- | --- | --- | --- | --- | --- | --- | --- | --- | --- | --- | --- | --- | --- | --- | --- |
| Sagittal corneal refractive power | | | | | | | | | | | | | | | | |
| k1 | | 0.00±0.00 | | 0.00±0.00 | | 0.00±0.00 | | 0.00±0.00 | | N/A | | 0.00±0.00 | | 0.00±0.00 | | N/A |
| k2 | | -0.04±0.06 | | -0.01±0.05 | | -0.06±0.09 | | -0.06±0.09 | | 0.76 | | -0.11±0.17 | | -0.11±0.13 | | 0.83 |
| k3 | | -0.09±0.12 | | -0.04±0.09 | | -0.14±0.21 | | -0.15±0.19 | | 0.93 | | -0.22±0.39 | | -0.19±0.26 | | 0.69 |
| k4 | | -0.14±0.17 | | -0.07±0.12 | | -0.20±0.32 | | -0.12±0.29 | | 0.27 | | -0.27±0.49 | | -0.05±0.36 | | 0.05 |
| k5 | | -0.20±0.23 | | -0.12±0.17 | | -0.35±1.02 | | 0.00±0.39 | | 0.03 | | -0.14±0.53 | | 0.24±0.42 | | 0.003* |
| k6 | | -0.28±0.25 | | -0.17±0.19 | | -0.27±1.01 | | 0.23±0.47 | | 0.002* | | 0.23±0.58 | | 0.72±0.41 | | <0.001* |
| k7 | | -0.40±0.28 | | -0.28±0.23 | | 0.05±0.45 | | 0.53±0.56 | | <0.001* | | 0.78±0.69 | | 1.17±0.49 | | 0.01 |
| k8 | | -0.57±0.31 | | -0.43±0.27 | | 0.32±0.52 | | 0.81±0.63 | | 0.001* | | 1.41±0.71 | | 1.41±0.69 | | 0.99 |
| Total corneal refractive power | | | | | | | | | | | | | | | | |
| k1 | 0.00±0.00 | | 0.00±0.00 | | 0.00±0.00 | | 0.00±0.00 | | N/A | | 0.00±0.00 | | 0.00±0.00 | | N/A | |
| k2 | 0.01±0.07 | | 0.02±0.06 | | -0.04±0.12 | | -0.06±0.09 | | 0.52 | | -0.07±0.22 | | -0.08±0.16 | | 0.84 | |
| k3 | 0.04±0.13 | | 0.07±0.11 | | -0.07±0.25 | | -0.08±0.19 | | 0.89 | | -0.13±0.45 | | -0.05±0.35 | | 0.44 | |
| k4 | 0.12±0.19 | | 0.18±0.15 | | -0.05±0.39 | | 0.02±0.30 | | 0.43 | | 0.04±0.60 | | 0.29±0.46 | | 0.07 | |
| k5 | 0.24±0.25 | | 0.33±0.21 | | 0.07±0.48 | | 0.29±0.41 | | 0.05 | | 0.46±0.65 | | 0.99±0.47 | | <0.001* | |
| k6 | 0.42±0.29 | | 0.52±0.25 | | 0.34±0.52 | | 0.77±0.50 | | 0.001* | | 1.37±0.70 | | 2.05±0.49 | | <0.001* | |
| k7 | 0.60±0.32 | | 0.73±0.28 | | 0.81±0.55 | | 1.40±0.57 | | <0.001* | | 2.66±0.81 | | 3.27±0.57 | | <0.001* | |
| k8 | 0.81±0.36 | | 0.79±0.27 | | 1.50±0.59 | | 2.12±0.65 | | <0.001* | | 4.35±0.91 | | 4.51±0.83 | | 0.44 | |

* p<0.006=significantly different
