# Supplementary material for: Comparative study of wave-front aberration and corneal Asphericity after SMILE and LASEK for myopia: a short and long term study
Source: BMC Ophthalmol. 2019 Mar 20;19:80. doi: 10.1186/s12886-019-1084-3 (PMC6425702; doi:10.1186/s12886-019-1084-3)
Supplement: Supplementary file 3 — Comparison of change in wave-front aberration in the two groups post surgery. This table shows the comparison of the change in wave-front aberration (postoperative-preoperative) between SMILE and LASEK post surgery. (DOCX 18 kb) [file 12886_2019_1084_MOESM3_ESM.docx]

Additional file 3. Comparison of change in wave-front aberration in the two groups post surgery

|  | SMILE 3m-0 | LASEK 3m-0 | *p* value | SMILE 3y-0 | LASEK 3y-0 | *p* value | SMILE 3y-3m | LASEK 3y-3m | *p* value |
| --- | --- | --- | --- | --- | --- | --- | --- | --- | --- |
| Vertical coma | 0.27±0.42 | 0.60±0.57 | 0.01* | 0.06±0.42 | 0.58±0.58 | <0.001* | -0.21±0.62 | -0.02±0.82 | 0.31 |
| Horizontal coma | 0.46±0.59 | 0.36±0.71 | 0.55 | 0.22±0.62 | 0.32±0.68 | 0.55 | -0.24±0.79 | -0.04±0.62 | 0.28 |
| Vertical trefoil | -0.01±0.24 | 0.10±0.31 | 0.11 | -0.02±0.35 | 0.01±0.31 | 0.70 | -0.01±0.33 | -0.09±0.39 | 0.39 |
| Horizontal trefoil | 0.13±0.26 | 0.06±0.43 | 0.42 | -0.04±0.23 | 0.00±0.46 | 0.63 | -0.17±0.31 | -0.06±0.36 | 0.17 |
| SA | -0.07±0.30 | -0.41±0.40 | <0.001* | 0.04±0.29 | -0.22±0.47 | 0.009* | 0.11±0.39 | 0.19±0.51 | 0.52 |
| Coma | 0.25±0.58 | 0.39±0.74 | 0.43 | 0.22±0.62 | 0.38±0.68 | 0.31 | -0.04±0.71 | -0.01±0.77 | 0.86 |
| Trefoil | -0.03±0.25 | 0.01±0.35 | 0.56 | 0.00±0.24 | -0.01±0.31 | 0.80 | 0.03±0.32 | -0.03±0.34 | 0.45 |
| HOA | 0.10±0.16 | 0.24±0.20 | 0.004* | 0.19±0.23 | 0.26±0.17 | 0.19 | 0.08±0.21 | 0.02±0.18 | 0.18 |

SA: Spherical aberration

HOA: Higher order aberration

3m-0: the change of aberration at 3 months post operation from preoperational

3y-0: the change of aberration at 3 years post operation from preoperational

3y-3m: the change of aberration at 3 years post operation from 3 months post operation

*p<0.05 significantly different
